# High-Pass Filter Characteristics of the Baroreflex – A Comparison of Frequency Domain and Pharmacological Methods

**DOI:** 10.1371/journal.pone.0079513

**Published:** 2013-11-14

**Authors:** Istvan Bonyhay, Marcelo Risk, Roy Freeman

**Affiliations:** 1 Department of Neurology, Beth Israel Deaconess Medical Center, Harvard Medical School, Boston, Massachusetts, United States of America; 2 Instituto Tecnologico de Buenos Aires (ITBA) and Consejo Nacional de Investigaciones Cientificas y Tecnicas (CONICET), Buenos Aires, Argentina; University of Adelaide, Australia

## Abstract

Pharmacological methods to assess baroreflex sensitivity evoke supra-physiological blood pressure changes whereas computational methods use spontaneous fluctuations of blood pressure. The relationships among the different baroreflex assessment methods are still not fully understood. Although strong advocates for each technique exist, the differences between these methods need further clarification. Understanding the differences between pharmacological and spontaneous baroreflex methods could provide important insight into the baroreflex physiology. We compared the modified Oxford baroreflex gain and the transfer function modulus between spontaneous RR interval and blood pressure fluctuations in 18 healthy subjects (age: 39±10 yrs., BMI: 26±4.9). The transfer function was calculated over the low-frequency range of the RR interval and systolic blood pressure oscillations during random-frequency paced breathing. The average modified Oxford baroreflex gain was lower than the average transfer function modulus (15.7±9.2 ms/mmHg vs. 19.4±10.5 ms/mmHg, *P<0.05*). The difference between the two baroreflex measures within the individual subjects comprised a systematic difference (relative mean difference: 20.7%) and a random variance (typical error: 3.9 ms/mmHg). The transfer function modulus gradually increased with the frequency within the low-frequency range (LF), on average from 10.4±7.3 ms/mmHg to 21.2±9.8 ms/mmHg across subjects. Narrowing the zone of interest within the LF band produced a decrease in both the systematic difference (relative mean difference: 0.5%) and the random variance (typical error: 2.1 ms/mmHg) between the modified Oxford gain and the transfer function modulus. Our data suggest that the frequency dependent increase in low-frequency transfer function modulus between RR interval and blood pressure fluctuations contributes to both the systematic difference (bias) and the random variance (error) between the pharmacological and transfer function baroreflex measures. This finding suggests that both methodological and physiological factors underlie the observed disagreement between the pharmacological and the transfer function method. Thus both baroreflex measures contribute complementary information and can be considered valid methods for baroreflex sensitivity assessment.

## Introduction

The baroreflex provides prompt reflex changes in heart rate and peripheral resistance, thereby buffering the blood pressure perturbations that occur in response to physiological or environmental provocations. Baroreflex sensitivity, defined as the reflex changes in heart period (RR interval) in response to changes in blood pressure, is the most frequently used characteristic of the baroreflex. Decreased baroreflex sensitivity is a feature of several disorders of the cardiovascular [1]–[3] and autonomic nervous system [4]–[6]. Baroreflex dysfunction is also a prognostic factor in several cardiovascular diseases [3], [7]–[13]. Estimation of baroreflex sensitivity is therefore an important component of autonomic and cardiovascular research and may have serious clinical implications. Although several techniques for baroreflex assessment are available and are widely used, there is no gold standard method for baroreflex assessment [14], [15].

Pharmacological methods to assess baroreflex sensitivity evoke supra-physiological blood pressure change as the input signal and the subsequent change in RR interval is analysed as function of blood pressure [16]–[18]. In contrast, computational methods such as the sequence and spectral methods use the much smaller spontaneous variations of blood pressure and RR interval for baroreflex assessment [19]–[22]. While large pressure changes evoked by pharmacological methods clearly cause baroreflex engagement, studies have also confirmed the role of the baroreflex in the interaction between spontaneous blood pressure and RR interval oscillations [23], [24]. Reports have also suggested that the low-frequency spontaneous RR interval changes are predominantly mediated by the baroreflex whereas the high-frequency RR interval and blood pressure oscillations may have concurrent mechanical and neural mechanisms [11], [23], [25]–[28].

Despite the extensive use of pharmacological and spontaneous baroreflex methods to assess baroreflex sensitivity, the relationship between these methods is not fully elucidated. Previous studies comparing pharmacological and spontaneous baroreflex measures showed moderate [29]–[32] or high [19], [33] linear association between the two methods but the pharmacological and spontaneous baroreflex measures generally had a weak agreement within the individual subjects. Although no systematic investigation has been performed, it is likely that both methodological and physiological factors play a role in the weak agreement between the pharmacological and spontaneous baroreflex methods [22].

The difference between two measurements comprises a systematic and a random factor [34]–[37]. The systematic difference or bias is a general trend for measurements to be different in a particular direction and usually originates from methodological or treatment effects, whereas the random variance or error is caused by unpredictable biological and technical variability that occurs between the measurements.

In the present study we compared two frequently used techniques for baroreflex assessment, the RR interval response to sequential pharmacological manipulation of blood pressure (the modified Oxford method) [38] and the transfer function analysis between the spontaneous RR interval and blood pressure oscillations [19], [22]. Although it is recognized that the modulus of the transfer function between spontaneous RR interval and blood pressure oscillations, the measure of baroreflex sensitivity, shows frequency dependency [19], [39], and even within the low-frequency band the modulus increases as function of frequency [40], [41], the role played by this phenomenon in producing the difference between the modified Oxford and the transfer function assessment methods has not been analysed.

We sought to elucidate the methodological and physiological factors that underlie the weak agreement between the pharmacological and spontaneous baroreflex measures within the individual subjects. We hypothesized that the frequency dependence of the modulus of the transfer function between spontaneous RR interval and blood pressure was a major contributor to the difference between the modified Oxford and the transfer function baroreflex assessment methods.

## Subjects and Methods

### Subjects

Eighteen healthy subjects (12 females, 6 males) participated in the study. All subjects were free from any acute illness or chronic disease. The study was performed in accordance with the Declaration of Helsinki of the World Medical Association and the protocol was approved by the Institutional Review Board of the Beth Israel Deaconess Medical Center. All subjects provided written informed consent prior to the study.

### Protocol

Subjects were studied in supine position, in the morning after a light breakfast. Heart rate, RR interval, respiration and blood pressure were monitored throughout the study. Respiratory pattern was recorded with a two-belt chest-abdomen inductance plethysmograph (Respitrace Ambulatory Monitoring). Blood pressure was continuously measured on the finger by Finapres (Model 2300, Ohmeda) and intermittently measured with oscillometry (Dinamap). On consecutive days, baroreflex sensitivity was estimated by the modified Oxford technique and by the analysis of transfer function between spontaneous RR interval and blood pressure fluctuations.

### Baroreflex sensitivity assessment

**Figure 1:**
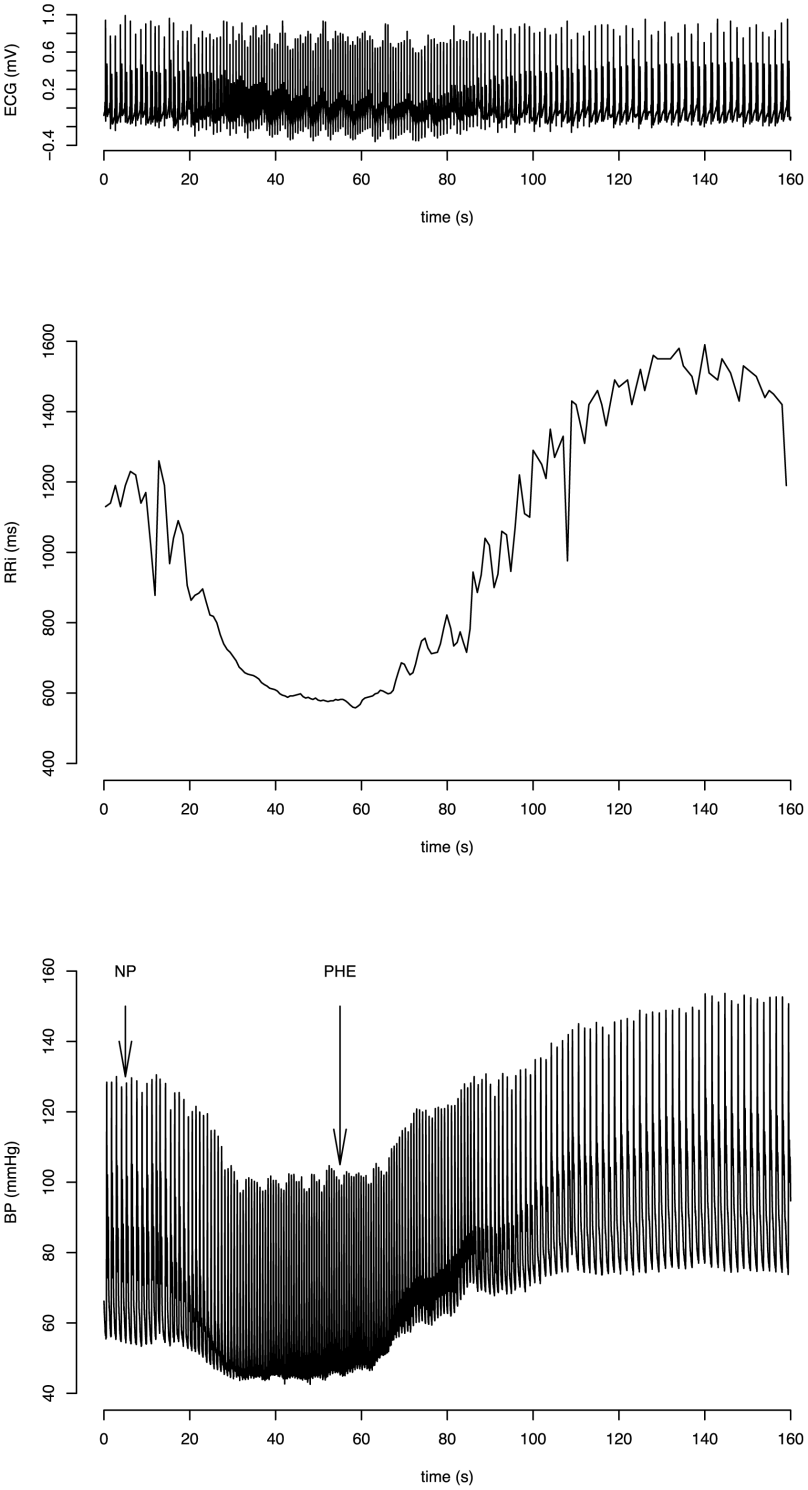
Modified Oxford baroreflex test. ECG, RR interval and blood pressure recording during the modified Oxford baroreflex test in a representative subject. Arrows indicate sodium nitroprusside (NP) and phenylephrine (PHE) bolus injections. Blood pressure fall is followed by a blood pressure rise in response to the bolus injections.

To perform the modified Oxford test, an intravenous catheter was inserted into an antecubital vein for drug administration. After a resting period of 30 min, a 5-min baseline recording was made and then followed by the baroreflex test: Sequential administration of bolus injections of 100 µg sodium nitroprusside and of 150 µg phenylephrine hydrochloride produced a drop in pressure of ∼15 mmHg below baseline followed by pressure rise of ∼15 mmHg above baseline (Figure 1) [38]. We performed the baroreflex test at least twice with a 15-minute recovery period between trials to allow heart rate and blood pressure to return to the normal values.

**Figure 2:**
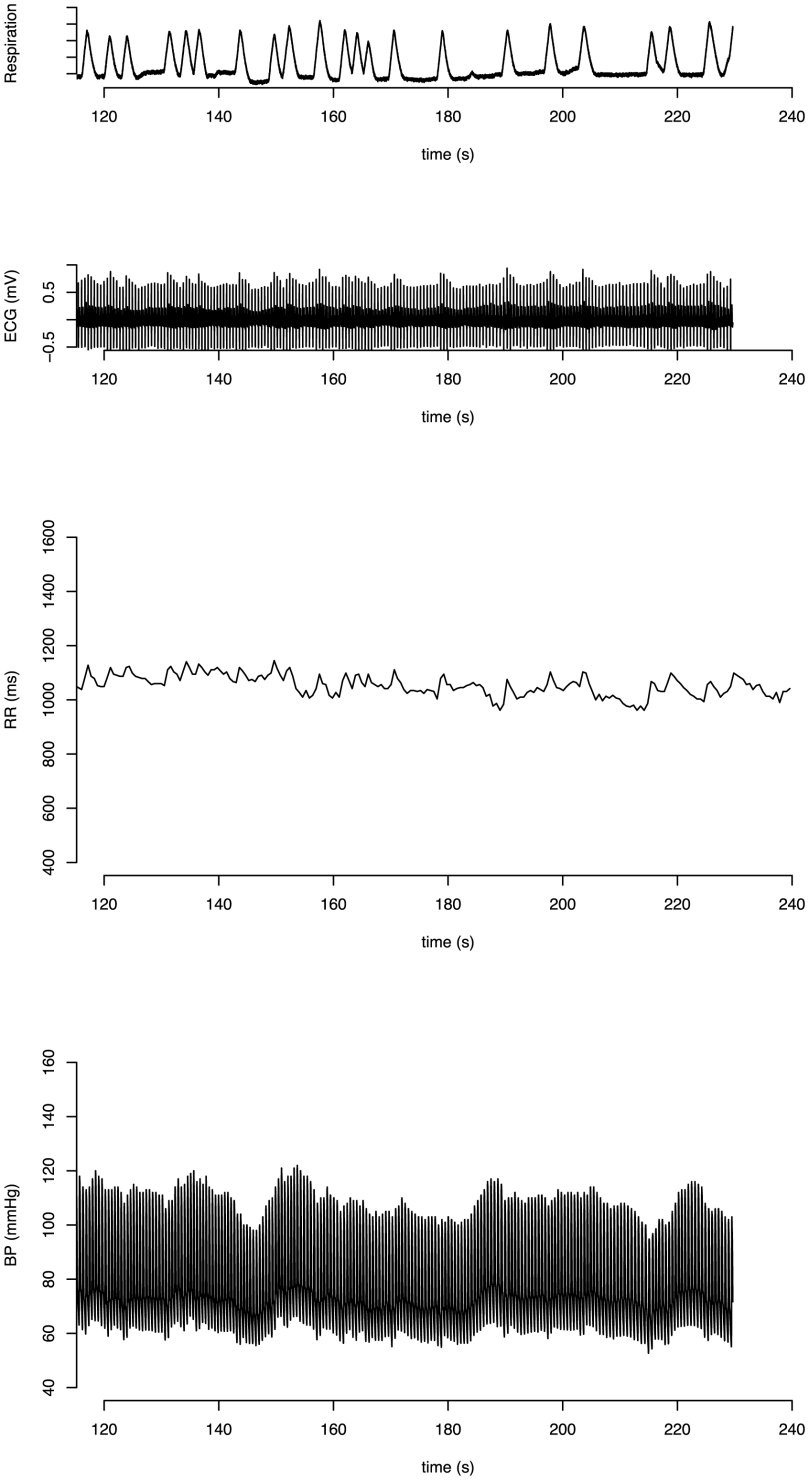
Random-frequency paced breathing. Respiration, ECG, RR interval and blood pressure recording during the random-frequency breathing protocol in a representative subject. Random-frequency breathing was used to broaden the frequency content of the respiratory signal for transfer function analysis.

For spontaneous baroreflex assessment, we used the transfer function between RR interval and blood pressure oscillations over the low-frequency band as it is thought to be predominantly determined by the baroreflex. The transfer function method has been described in detail previously [39], [42]. Briefly, after a 30-min resting period, the subjects performed a 7-min random-frequency paced breathing protocol in which a breath was initiated with each tone of a series of auditory cues spaced at irregular intervals (Figure 2). The median breathing frequency was 0.26±0.03 Hz with an inter-quartile range of 0.26±0.06 Hz, averaged across the subjects. To minimize paced breathing related stress, the subjects were trained to breathe in response to auditory cues prior to the test. Also, to minimize the discomfort and hyperventilation, the subjects were allowed to comfortably control the depth and shape of each breath throughout the breathing protocols.

### Data analysis

**Figure 3:**
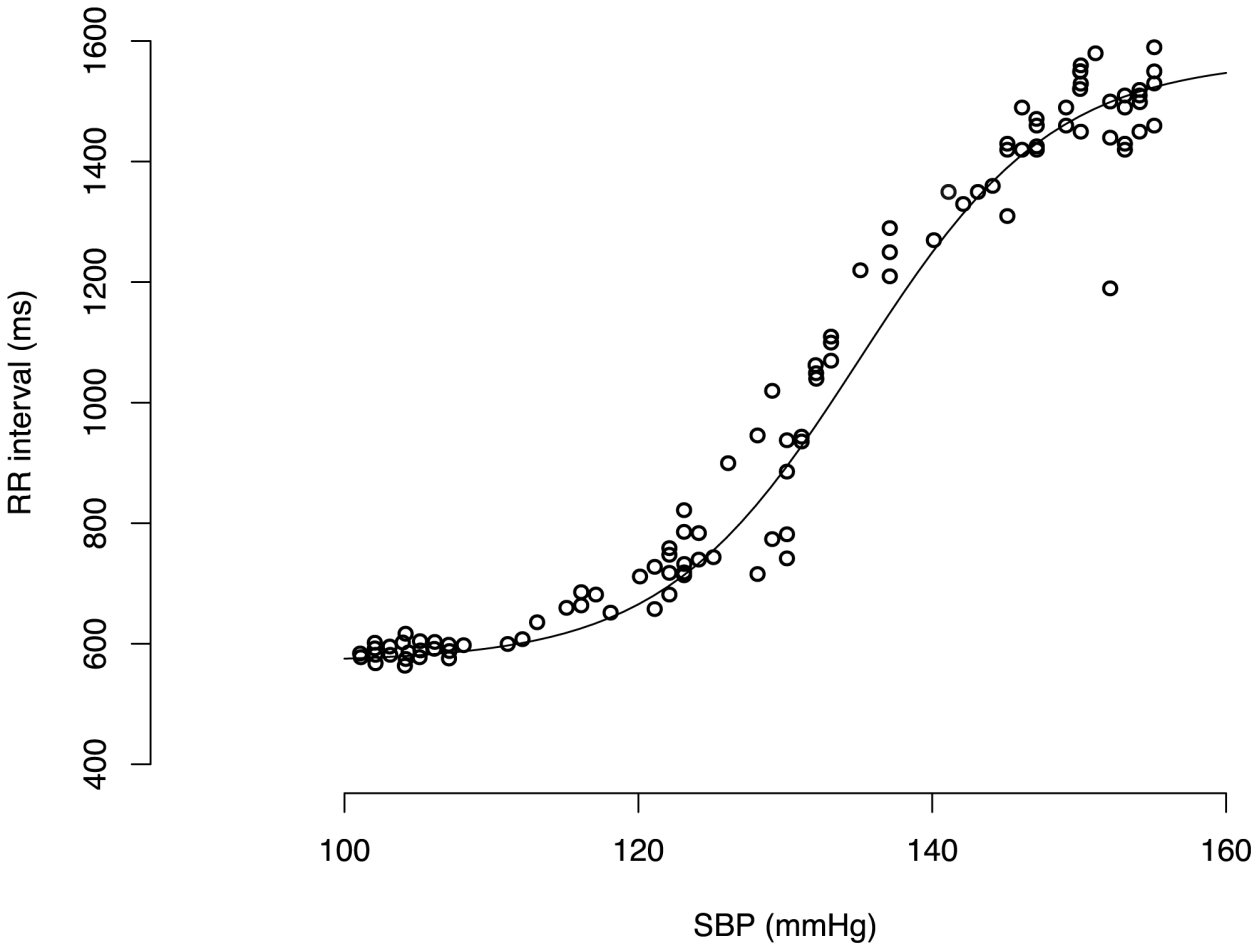
Modified Oxford baroreflex assessment in a representative subject. For baroreflex sensitivity assessment RR interval is plotted as a function of systolic blood pressure (SBP) between the lowest and the peak pressure values. The linear part of the sigmoid function describes the baroreflex sensitivity (see Methods).

Baroreflex sensitivity in the modified Oxford method was assessed by beat-to-beat RR interval plotted as a function of systolic blood pressure between the lowest and the peak pressure values (Figure 3). In 88% of subjects this revealed the entire sigmoid nature of arterial baroreflex [18], [43]. After extracting the saturation and threshold regions, the slope of the linear part of regression between RR interval and systolic blood pressure provided the measure of baroreflex sensitivity [17]. Only regressions with correlation coefficients r>0.7 were accepted.

For transfer function analysis the Blackman-Tukey method [44] was used to calculate the power spectrum of RR interval and systolic blood pressure. The relevant segments for spectral analysis were 341 seconds (1024 samples at 3 Hz). The frequency range 0.04−0.15 Hz was used to define the low-frequency band. The transfer function between RR interval and systolic blood pressure oscillations was calculated using the cross-spectra method [26], [42], [45]. The transfer function modulus was estimated as the mean value in the low-frequency range including only the segments with coherence >0.5.

**Figure 4:**
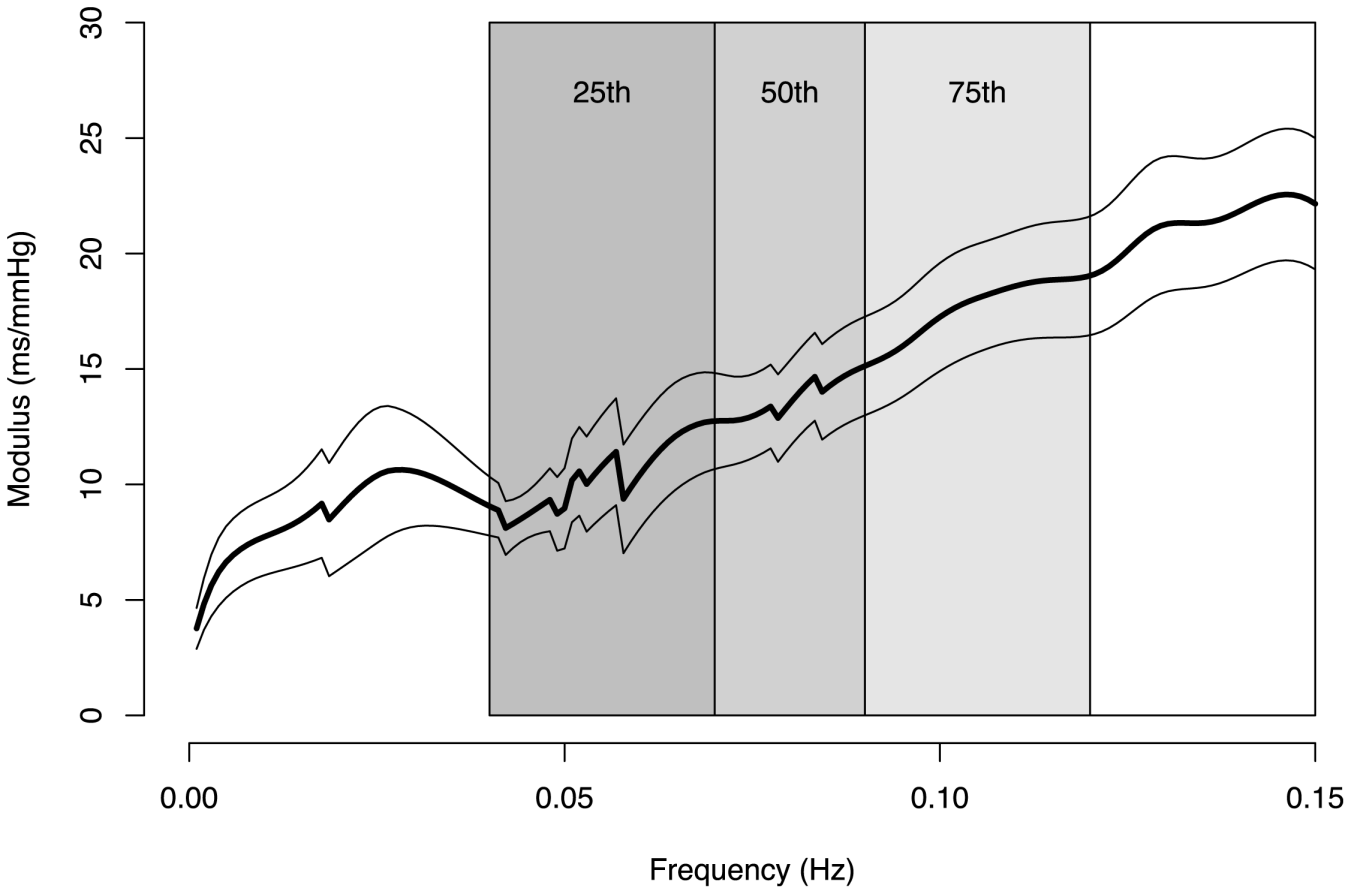
Transfer function modulus curve in the low-frequency region averaged for all subjects. Thick line displays average values, thin lines display standard deviation of transfer function modulus. Shaded areas represent the segments calculated according to the 25^th^, 50^th^ and 75^th^ percentiles of the transfer function modulus (see Methods).

To account for the frequency dependent increase in the modulus of the transfer function, the transfer function within the low-frequency band was subdivided into segments encompassing the 25^th^, 50^th^, 75^th^ percentiles of the area under the modulus curve (Figure 4). The mean transfer function modulus was calculated for each of these segments within the low-frequency band.

### Statistical analysis

The relation between the modified Oxford baroreflex gain and the transfer function modulus was determined with the Pearson correlation coefficient. Paired t-test was used to compare blood pressure changes and baroreflex measures within the subjects.

To estimate the systematic difference (bias) and the random variance (error) between the modified Oxford baroreflex gain and the transfer function modulus, we used the concept for the measures of reliability described by Hopkins [34] and Bland and Altman [35].

**Figure 5:**
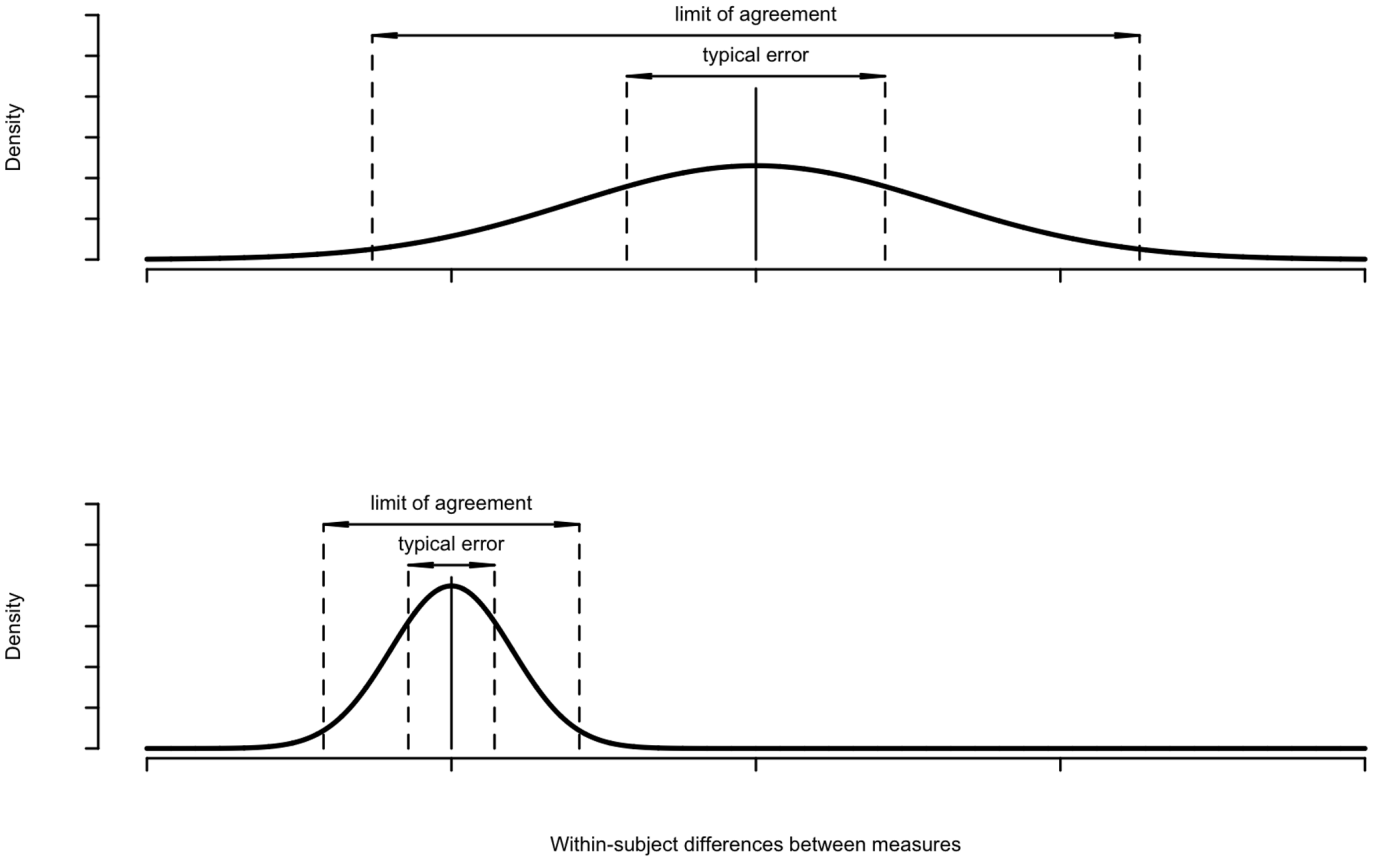
Systematic difference and random variance. Representation of the calculation of the systematic difference and random variance between modified Oxford and transfer function baroreflex measures. Mean of the within-subject difference scores denotes systematic difference (middle line). Standard deviation of within-subject difference scores divided by √2 denotes typical error. The 95% range of within-subject difference scores denotes limit of agreement. Top panel: Large systematic difference and large random variance between two measures. Bottom panel: Small systematic difference and small random variance between two measures.

The mean of the within-subject differences between the modified Oxford gain and the transfer function modulus denotes the systematic difference (bias) while the distribution of the within-subject differences (typical error and limit of agreement) between the two measures denotes the random variance between the two methods. Figure 5 illustrates the reliability concept. The figure displays two representative situations, one with large systematic difference and large random variance between two methods and one with small systematic difference and small random variance between two methods (Figure 5).

The typical error between the two baroreflex measures is calculated as the standard deviation (SD) of the within-subject differences divided by √2 [34]. The limit of agreement between the two baroreflex measures is calculated as the 95% likely range of the within-subject differences [34].

In order to reduce any proportional effect of baroreflex sensitivity on the within-subject differences between the two measures, and to express both the systematic difference (bias) and the random variance between the methods in relative terms, the systematic difference and random variance were also calculated from the logarithmically transformed data.

The systematic difference was expressed as the relative difference (%) [34] using the formula:
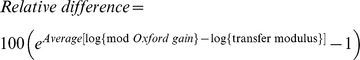



The random variance was also expressed as coefficient of variation (%) [34]using the formula:
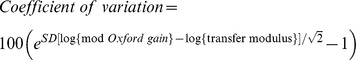



## Results

### Demographics and baseline parameters

The mean age of the subjects was 39±10 years while the BMI of the subjects was 26±4.9 kg/m^2^. The average baseline resting systolic blood pressure was 122±12 mmHg; diastolic blood pressure was 70±10 mmHg; and RR interval and heart rate were 1074±159 ms and 57±7 bpm.

### Blood pressure change

The range of blood pressure changes for baroreflex assessment was significantly different between the modified Oxford and the transfer function methods. Using the modified Oxford method, the range of systolic blood pressure change was 47±18 mmHg while using the transfer function method, the range of systolic blood pressure change was 24±9 mmHg (P<0.05).

### Modified Oxford gain and transfer function modulus

There was strong correlation between the modified Oxford baroreflex gain and the transfer function modulus among the subjects (r = 0.85). Despite their correlation, however, the two measures of baroreflex sensitivity differed considerably within individual subjects. On average among the subjects, the transfer function modulus was higher than the modified Oxford baroreflex gain (19.4±10.5 vs. 15.7±9.2, P<0.05).

**Figure 6:**
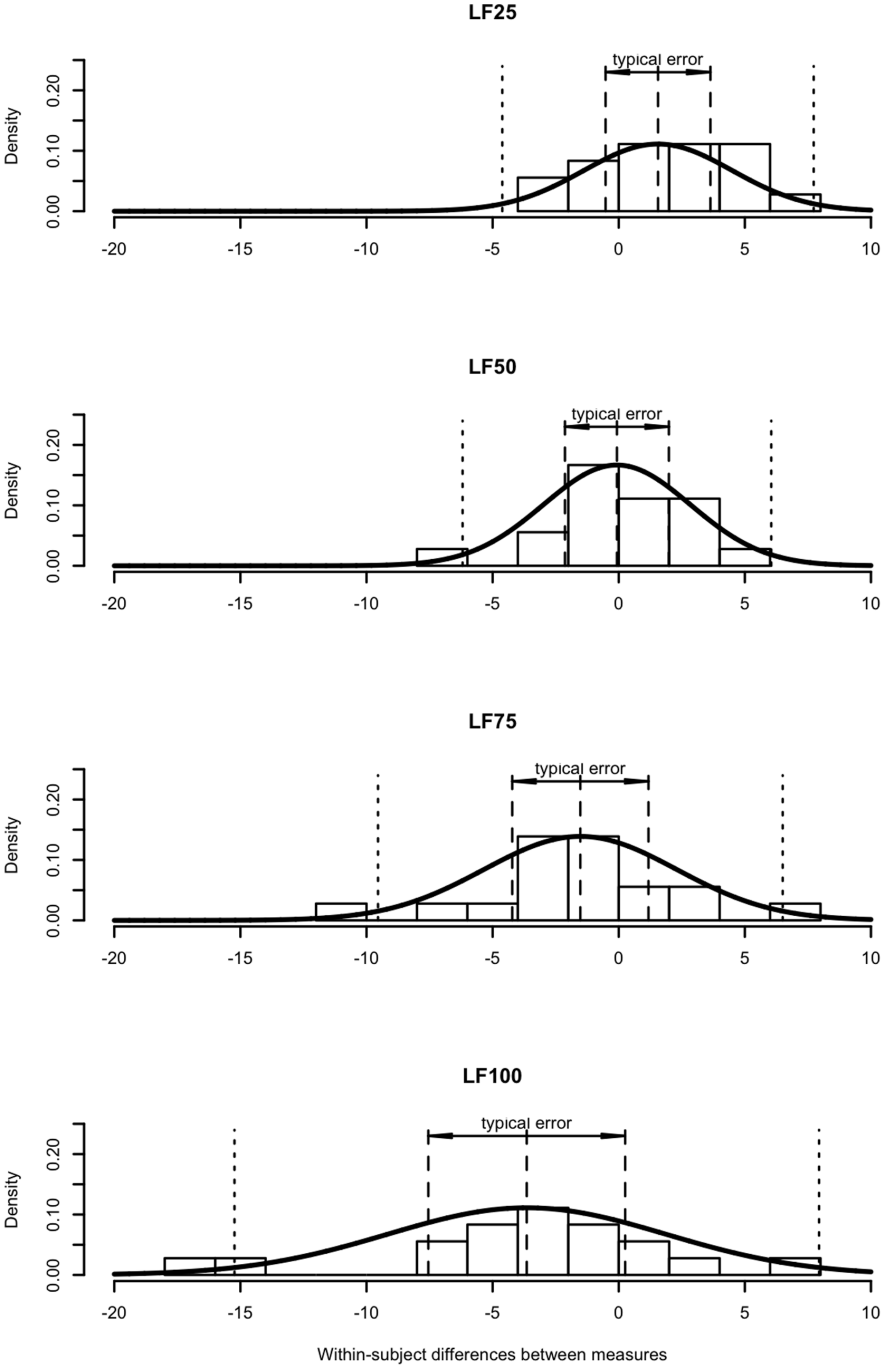
Systematic difference and random variance between the modified Oxford gain and the transfer function moduli. Systematic difference and random variance between modified Oxford baroreflex gain and the transfer function modulus calculated from the different segment of the low-frequency range. LF25, LF50, LF75 denote the segments in which the transfer function modulus was calculated for the analysis. LF100 denotes the entire low-frequency range between 0.04–0.15 Hz. Dashed lines represent mean difference and typical error. Dotted lines represent limit of agreement.

**Figure 7:**
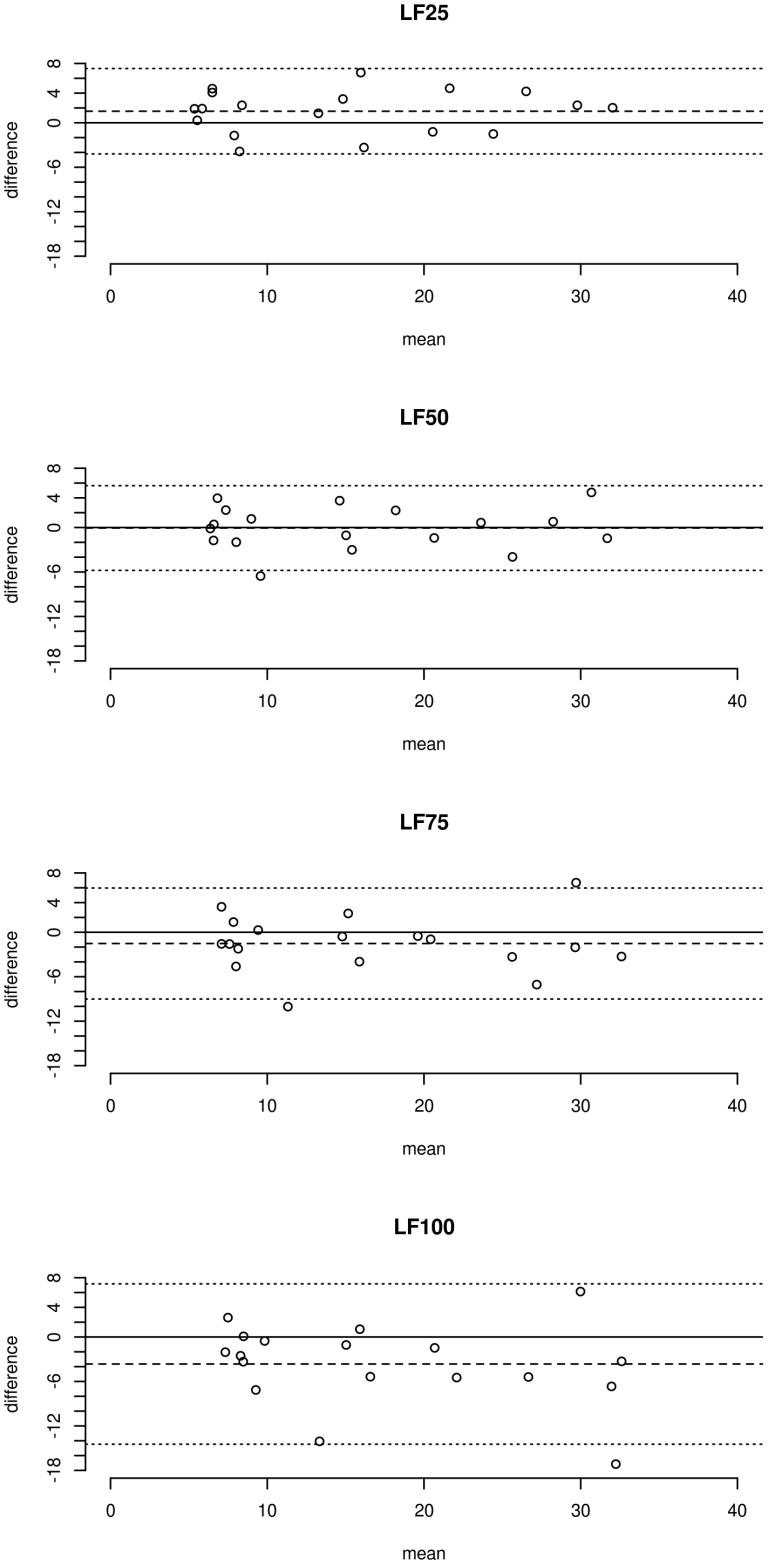
Bland-Altman plots of differences between modified Oxford baroreflex gain and transfer function moduli (y axis) and mean of the two measures (x axis). Dashed line denotes mean difference; dotted line denotes limit of agreement (range within which an individual's difference scores would fall 95% of the time).

The mean difference between the transfer function modulus and the modified Oxford gain was 3.6±5.5 ms/mmHg; the mean relative difference between the two baroreflex measures was 20.7%. The typical error between the transfer function modulus and the modified Oxford baroreflex gain was 3.9 ms/mmHg, the limit of agreement between the two baroreflex measures was 10.8 ms/mmHg. The coefficient of variation between the modified Oxford baroreflex gain and the transfer function modulus was 28.6% (see Figure 6 and Figure 7).

In comparison, the typical error between two consecutive modified Oxford baroreflex gains was 6.7 ms/mmHg or 19.7% expressed as coefficient of variation.

### Frequency dependent increase of transfer function modulus

Figure 4 shows the transfer function modulus as function of frequency over the low-frequency range, averaged for all subjects. The figure indicates that the transfer function modulus gradually increases with the frequency. The average increase of the transfer function modulus within the low-frequency band was 13.7±8.2 ms/mmHg across the subjects.

Figure 4 also illustrates the segmentation of the transfer function modulus curve (see Methods).

The systematic difference and the random variance (typical error and limit of agreement) between the modified Oxford gain and the moduli of transfer function calculated over the different segments of the low-frequency transfer function are displayed in Figure 6 and Figure 7 and summarized in Table 1 and Table 2. Both the systematic difference and the random variance between the modified Oxford gain and the transfer function modulus decreased when the modulus was calculated from lower (75^th^ and 50^th^ percentile) segments of the transfer function modulus curve (Figure 6–7 and Table 1–2). The modulus, calculated over the 25^th^ percentile segment of the modulus curve, was lower than the modified Oxford gain and the random variance between the two measures increased slightly.

**Table 1:** Systematic difference and random variance between modified Oxford baroreflex gain and transfer function modulus.

RAW DATA	Modified Oxford gain versus			
	LF100	LF75	LF50	LF25
Mean difference [ms/mmHg]	−3.6 (−6.4, −0.9)	−1.5 (−3.4, 0.4)	−0.07 (−1.5, 1.4)	1.6 (3.0, 0.1)
Typical error [ms/mmHg]	3.9 (2.9, 5.8)	2.7 (2.0, 4.0)	2.1 (1.5, 3.0)	2.1 (1.6, 3.1)
Limit of agreement [ms/mmHg]	10.8	7.5	5.7	5.8

Values were calculated from raw data. Transfer function was obtained from random-frequency breathing. The moduli were calculated over the entire low-frequency transfer function and over the different segments of the low-frequency transfer function (see Methods).

Values are expressed as means (95% Confidence Interval). LF100 denotes the transfer function modulus calculated over the entire low-frequency band. LF75, LF50 and LF25 denote the transfer function modulus obtained from the respective percentile segments of the low-frequency transfer function (see Methods).

**Table 2:** Systematic difference and random variance between modified Oxford baroreflex gain and transfer function modulus.

LOGARITHMIC DATA	Modified Oxford gain versus			
	LF100	LF75	LF50	LF25
Relative difference [%]	−20.7 (−31.9, −2.9)	−10.9 (−23.4, 5.0)	0.5 (−12.4,15.3)	15.9 (0.9, 36.3)
Coefficient of variation [%]	28.6 (23.9, 53.5)	25.1 (20.8, 45.8)	21.6 (17.6, 38.2)	23.8 (19.6, 42.9)

Values were calculated from logarithmically-transformed data. Transfer function was obtained from random-frequency breathing. The moduli were calculated over the entire low-frequency transfer function and over the different segments of the low-frequency transfer function (see Methods).

Values are expressed as means (95% Confidence Interval). LF100 denotes the transfer function modulus calculated over the entire low-frequency band. LF75, LF50 and LF25 denote the transfer function modulus obtained from the respective percentile segments of the low-frequency transfer function (see Methods).

### Comparison of transfer function modulus segments

Table 3 and Table 4 show the systematic difference and the random variance between the low-frequency modulus calculated over the entire low-frequency band (LF100) and the moduli calculated over the 75^th^ (LF75), 50^th^ (LF50) and 25^th^ (LF25) segments. Both the systematic difference and the random variance between the modulus from the entire low-frequency band (LF100) and the modulus from a lower segment increased when the modulus was calculated from a lower percentile segment of the transfer function modulus curve. The corresponding frequency limits for the 25^th^, 50^th^ and 75^th^ percentile segments of the low-frequency transfer function modulus were 0.07±0.02 Hz, 0.09±0.02 Hz and 0.12±0.02 Hz, averaged across the subjects.

**Table 3:** Systematic difference and random variance between the transfer function modulus calculated over the entire low-frequency band and the moduli calculated over the segments of the low-frequency transfer function.

RAW DATA	LF100 versus		
	LF75	LF50	LF25
Mean difference [ms/mmHg]	2.1 (0.9, 3.4)	3.6 (1.7, 5.4)	5.2 (2.6, 7.8)
Typical error [ms/mmHg]	1.7 (1.3, 2.6)	2.6 (1.9, 3.8)	3.7 (2.7, 5.5)
Limit of agreement [ms/mmHg]	4.8	7.2	10.2

Values were calculated from raw data. Values are expressed as means (95% Confidence Interval). LF75, LF50 and LF25 denote the transfer function modulus obtained from the respective percentile segments of the low-frequency transfer function (see Methods).

**Table 4:** Systematic difference and random variance between the transfer function modulus calculated over the entire low-frequency band and the moduli calculated over the segments of the low-frequency transfer function.

LOGARITHMIC DATA	LF100 versus		
	LF75	LF50	LF25
Relative difference [%]	11.0 (6.5, 14.1)	20.3 (11.0, 25.2)	31.6 (15.2, 37.3)
Coefficient of variation [%]	6.2 (4.8, 9.7)	13.1 (10.4, 21.8)	23.8 (19.6, 43.0)

Values were calculated from logarithmically-transformed data. Values are expressed as means (95% Confidence Interval). LF75, LF50 and LF25 denote the transfer function modulus obtained from the respective percentile segments of the low-frequency transfer function (see Methods).

## Discussion

In the present study we analysed the difference between the modified Oxford pharmacological baroreflex gain and the transfer function modulus of spontaneous low-frequency RR interval and blood pressure oscillations in healthy subjects. The main findings are: 1) the difference between the modified Oxford baroreflex gain and the low-frequency transfer function modulus between RR interval and blood pressure oscillations comprises a systematic difference (bias) and a random variance (error); 2) the systematic difference between the modified Oxford baroreflex gain and the low-frequency transfer function modulus decreases when the region within the low-frequency band is constricted to the lower segments; and 3) the random variance (error) between the modified Oxford baroreflex gain and the low-frequency transfer function modulus decreases as the systematic difference (bias) between the two methods is decreased.

Although the modified Oxford gain and the transfer function modulus correlate significantly across subjects, their values differ considerably within the individual subjects. This difference between the two baroreflex measures within the same subject has two components, the systematic difference, or bias and random variance or error [34], [36].

### Systematic difference

Consistent with prior studies, we show that the modified Oxford baroreflex gain is smaller than the LF transfer function modulus [46]. The significant difference in means between the modified Oxford baroreflex gain and the transfer function modulus across subjects represents the systematic difference between the two baroreflex measures and denotes methodological or treatment differences. There are several hypotheses to explain this systematic difference. These include: (1) The different features of open loop and closed loop system approach - the modified Oxford pharmacological baroreflex test represents an open loop approach of engaging the baroreflex system and thus the modified Oxford baroreflex gain represents only feedback relation between the RR interval and blood pressure. In contrast, the spontaneous baroreflex method represents a closed loop condition including both feedback and feed forward relations between RR interval and blood pressure. The interaction of the feed forward and feedback relations may change the measured baroreflex gain systematically [39], [43]. (2) Baroreflex resetting during blood pressure change - rapid resetting of the baroreflex during induced blood pressure changes could confound the measured baroreflex gain [43], [46]–[49]. Because the time course of the drug-induced blood pressure change is longer than that of the spontaneous pressure change, baroreflex resetting is more likely occur during the modified Oxford test [29], [43]. (3) The different relative influences of the sympathetic and parasympathetic systems evoked by the different methods – in the pharmacological method the blood pressure drop induced by the nitroprusside results in a rapid increase in sympathetic activity that lasts until the blood pressure returns to a normal level induced by the phenylephrine. The increased sympathetic activity, which is much less prominent during spontaneous decreases in pressure compared to drug-induced decreases in pressure, may have an attenuating effect on the measured baroreflex gain during the modified Oxford test [33]. (4) Direct cardiac effects - both the nitroprusside and phenylephrine used in the modified Oxford technique may have a direct effect on the heart, which could systematically modify the relationship between the RR interval and blood pressure. (5) The computation of baroreflex sensitivity along the modified Oxford baroreflex curve - fitting sigmoid or linear regression on the RR interval and blood pressure data, used in the calculation of the gain in the modified Oxford method, may also introduce a systematic difference in the measured baroreflex gain [18], [50].

Although we cannot exclude a contribution from these mechanisms, the present data suggest that a methodological factor in the transfer function analysis plays a major role in the systematic difference between the modified Oxford and the transfer function baroreflex measures. In accordance with other studies [29], [40], [41], we found that the transfer function modulus between blood pressure and RR interval oscillations gradually increased with the frequency within the low-frequency band (see Figure 4). Since the transfer function modulus is calculated as mean value over a defined frequency range, the frequency related increase in modulus may result in an overestimation of baroreflex sensitivity compared to the modified Oxford gain. Our results support this possibility. The calculation of transfer function modulus from the lower segments of the low-frequency modulus curve resulted in a significant reduction in the systematic difference between the transfer function modulus and the modified Oxford gain (see Figure 6–7 and Table 1–2).

The frequency dependent increase in transfer function modulus, that underlies the systematic difference between the two baroreflex methods, also highlights an important physiological characteristic of the baroreflex. The present data suggest that the blood pressure and RR interval change in the modified Oxford method corresponds to a low frequency RR interval and blood pressure fluctuation, while the transfer function method encompasses a broader frequency range, including higher frequency components of RR interval and blood pressure fluctuation that are under baroreflex control [24], [25]. The gradually increasing transfer function modulus as function of frequency indicates that the baroreflex operates as a high-pass filter, i.e., its gain increases as the rate of input signal increases [50]. Thus, the transfer function method allows the estimation of this frequency dependent characteristic of the baroreflex. This baroreflex feature has been documented earlier in animal studies [51], [52] and the rate sensitivity of baroreflex has also been demonstrated in humans [53].

The present data can also be viewed within the context of studies suggesting that the difference between the high-frequency and low-frequency spectral baroreflex estimates may in part be because a large portion of the RR variability in the high-frequency band is unrelated to pressure changes, i.e., not mediated by baroreflex [46], [49] and thus may artificially increase the transfer function estimate [54]. It is possible that the high-pass filter characteristic of baroreflex contributes to this observed difference between spectral indices derived in the high-frequency band and in the low-frequency band. We also note that, the modulus calculated from the lowest segment of the low-frequency range (see 25^th^ percentile results) was lower than the mean modified Oxford gain across subjects. These data suggest that the corresponding frequency range for the modified Oxford related blood pressure and RR interval change is around 0.09±0.02 Hz, which is defined by the 50^th^ percentile of the modulus curve.

### Random variance

The random variance or error between two measurements reflects methodological and biological variation occurring from measurement to measurement [34], [36]. Prior studies comparing baroreflex assessment methods typically found considerable random variance between the pharmacological and transfer function baroreflex measures which was usually reported as a large limit of agreement [29], [30], [43]. The large random variance between the modified Oxford gain and the low-frequency transfer function modulus that we observed in the present study is consistent with these earlier studies. However, in the present study we also observed that the random variance between the two baroreflex measures decreased when the systematic bias between the two methods was reduced (see Figure 6–7 and Table 1–2). The random variance (error) between the modified Oxford gain and the low-frequency transfer modulus decreased from ∼30% to ∼20% as the systematic difference was reduced too (Table 2). This ∼10% decrease in the random variance between the modified Oxford gain and the transfer function modulus was comparable to the random variance that we observed between the low-frequency modulus and the modulus from the lower transfer function segment (Table 4) – which reflects purely the effect of the increasing modulus. This suggests that the frequency dependent increase of transfer function modulus, which causes the systematic difference between the two methods, inflates the random variance between the two baroreflex assessment methods.

### Limitation

In the present study, we used the standard coherence criterion (>0.5) method [39] to calculate transfer function modulus over the low-frequency band. Recent studies, however, have suggested that the coherence function can overestimate the role of the baroreflex in the interaction between RR interval and arterial pressure variability due to the co-existence of feedback and feed forward mechanisms and the involvement of other non-baroreflex mechanisms in the synchronous changes of RR interval and blood pressure [49], [55], [56]. In the present study, we focused exclusively on low-frequency transfer function, and in agreement with the literature we assumed that the RR interval in our middle-aged healthy subjects was mainly driven by the blood pressure variations in the low-frequency range [48], [49], [56], [57].

## Conclusions

The present study provides a new insight into the comparison of pharmacological and frequency domain baroreflex methods by demonstrating that the difference between the modified Oxford baroreflex gain and the transfer function modulus of RR interval and blood pressure oscillations, at least in part, originates from the frequency dependent, high-pass filter characteristic of the baroreflex. While the modified Oxford method derives baroreflex gain by assessing the changes of RR interval in response to a single large transient biphasic blood pressure change, the transfer function between spontaneous blood pressure and RR interval oscillations assesses the baroreflex across a range of frequencies. Our data suggest that these two methods for baroreflex assessment provide complementary information on the baroreflex. The modified Oxford method assesses the entire baroreflex curve from threshold to saturation while the transfer function analysis allows the estimation of the frequency dependent characteristics of baroreflex. The differences between these methods of baroreflex assessment may have implications for the study of baroreflex dysfunction in autonomic and cardiovascular disorders.
